# ^18^F-FET MicroPET and MicroMRI for Anti-VEGF and Anti-PlGF Response Assessment in an Orthotopic Murine Model of Human Glioblastoma

**DOI:** 10.1371/journal.pone.0115315

**Published:** 2015-02-13

**Authors:** Mette Kjoelhede Nedergaard, Signe Regner Michaelsen, Thomas Urup, Helle Broholm, Henrik El Ali, Hans Skovgaard Poulsen, Marie-Thérése Stockhausen, Andreas Kjaer, Ulrik Lassen

**Affiliations:** 1 Department of Clinical Physiology, Nuclear Medicine & PET and Cluster for Molecular Imaging, Rigshospitalet and University of Copenhagen, Copenhagen, Denmark; 2 Department of Radiation Biology, The Finsen Center, Rigshospitalet, Copenhagen, Denmark; 3 Department of Neuropathology, Center of Diagnostic Investigation, Rigshospitalet, Copenhagen, Denmark; 4 Phase 1 Unit, Department of Oncology, The Finsen Center, Rigshospitalet, Copenhagen, Denmark; University of Udine, ITALY

## Abstract

**Background:**

Conflicting data exist for anti-cancer effects of anti-placental growth factor (anti-PlGF) in combination with anti-VEGF. Still, this treatment combination has not been evaluated in intracranial glioblastoma (GBM) xenografts. In clinical studies, position emission tomography (PET) using the radiolabeled amino acid O-(2-^18^F-fluoroethyl)-L-tyrosine (^18^F-FET) and magnetic resonance imaging (MRI) add complementary but distinct information about glioma growth; however, the value of ^18^F-FET MicroPET combined with MicroMRI has not been investigated preclinically. Here we examined the use of ^18^F-FET MicroPET and MicroMRI for evaluation of anti-VEGF and anti-PlGF treatment response in GBM xenografts.

**Methods:**

Mice with intracranial GBM were treated with anti-VEGF, anti-PlGF + anti-VEGF or saline. Bioluminescence imaging (BLI), ^18^F-FET MicroPET and T2-weighted (T2w)-MRI were used to follow tumour development. Primary end-point was survival, and tumours were subsequently analysed for Ki67 proliferation index and micro-vessel density (MVD). Further, PlGF and VEGFR-1 expression were examined in a subset of the xenograft tumours and in 13 GBM patient tumours.

**Results:**

Anti-VEGF monotherapy increased survival and decreased ^18^F-FET uptake, BLI and MVD, while no additive effect of anti-PlGF was observed. ^18^F-FET SUV_max_ tumour-to-brain (T/B) ratio was significantly lower after one week (114±6%, n = 11 vs. 143±8%, n = 13; p = 0.02) and two weeks of treatment (116±12%, n = 8 vs. 190±24%, n = 5; p = 0.02) in the anti-VEGF group as compared with the control group. In contrast, T2w-MRI volume was unaffected by anti-VEGF. Gene expression of PlGF and VEGFR-1 in xenografts was significantly lower than in patient tumours.

**Conclusion:**

^18^F-FET PET was feasible for anti-angiogenic response evaluation and superior to T2w-MRI; however, no additive anti-cancer effect of anti-PlGF and anti-VEGF was observed. Thus, this study supports use of ^18^F-FET PET for response evaluation in future studies.

## Introduction

It is widely accepted that angiogenesis is a fundamental process for tumour progression and metastasis. Vascular endothelial growth factor A (VEGF) is considered a major pro-angiogenic mediator in glioblastoma multiforme (GBM), the most common and aggressive type of primary brain tumours in adults [1]. VEGF signalling is primarily mediated through the receptors VEGFR-1 and VEGFR-2; although VEGFR-2 is the major receptor involved in angiogenesis [2]. Placental growth factor (PlGF) is a member of the VEGF family of growth factors. PlGF binds selectively to VEGFR-1 and its soluble isoform, termed sVEGFR-1 [3]. Under pathological conditions, such as cancer, the expression of PlGF is up-regulated and binding of PlGF to VEGFR-1 is in general considered as pro-angiogenic; however, the precise function of PlGF and VEGFR-1 in angiogenesis and tumour growth is still under debate [3–5]. PlGF is one of several growth factors that have been implicated in resistance to anti-angiogenic therapies [6]. Still, conflicting opinions exist on the value of neutralizing PlGF as a therapeutic target in oncology. Fischer *et al.* reported that anti-VEGF and anti-PlGF had an additive anti-tumour activity in several subcutaneous xenograft tumour models [6] and these results were later supported by others [7]. Conversely, other groups have found either no anti-tumour activity of anti-PlGF [4] or even a suppressive effect of PlGF on tumour growth and angiogenesis [8–11]. However, none of these studies have evaluated the anti-cancer activity of anti-PLGF in an intracranial GBM model. Furthermore, it has been demonstrated that the expression of VEGFR-1 in cancer cells could determine the efficacy of anti-PlGF treatment, a hypothesis that was suggested as a possible explanation for the conflicting data in the literature [12].

Imaging and response assessment of gliomas by conventional magnetic resonance imaging (MRI) is complicated [13, 14]. As we have demonstrated previously, positron emission tomography (PET) with the radiolabeled amino acid O-(2–^18^F-fluoroethyl)-L-tyrosine (^18^F-FET) is feasible for assessment of treatment response in an orthotopic xenograft model of GBM [15]. In patients with glioma, ^18^F-FET PET (compared to MRI alone) adds additional information about tumour growth [16–19]; however, these two modalities have not been combined and evaluated in an orthotopic xenograft model of GBM.

In the present study, we hypothesized that by combining anti-VEGF and anti-PlGF therapies it would be possible to obtain an additive anti-tumour effect in an orthotopic xenograft model of GBM. In addition, we hypothesized that the combination of T2w-MRI and ^18^F-FET MicroPET would give additional information about tumour growth and response to therapy.

## Materials and Methods

### Ethics Statement

This study was performed according to the Declaration of Helsinki and Danish legislation. The Scientific Ethical Committee for Copenhagen and Frederiksberg (KF-01–327718) approved the use of patient tissue for gene expression analysis and for establishment of the *in vitro* cell culture NGBM_CHP017p4 as previously described [20], and permissions were given from the Danish Data Protection Agency (2006–41–6979). Written informed consent was obtained from the patients. Animal care and all experimental procedures were performed under the approval of the Danish Animal Welfare Council (2013–15–2934–00064). Animal surgery and euthanasia using decapitation were performed under Hypnorm/Midazolam anaesthesia, and all efforts were made to minimize suffering.

### Cells and patient specimens

NGBM_CPH017p4 cells, having a stable expression of luciferase (GBM017_LUC), were used for xenograft generation. Establishment, maintenance and luciferase transduction has previously been described [15, 20]. Tumour specimens from 13 GBM patients obtained at primary surgery were randomly chosen and used for the gene expression analysis, and isolated RNA from human microvascular endothelial cells (HMVEC) was used as a positive control. The panel of patient tumours included the patient tumour (GBM017) used for establishment of the neurosphere cell culture NGBM_CPH017p4.

### Establishment of a human orthotopic GBM model and experimental design

Six weeks old NMRI (Naval Medical Research Institute) nude female mice were acquired from Taconic Europe (Lille Skensved, Denmark). Following a minimum of one week of acclimatization, mice were injected intracranially with ten µl cell suspension (100,000 cells) of GBM017_LUC as previously described [15]. Mice were injected with cells at week 0 and from week 3, weekly bioluminescence imaging (BLI), MRI and ^18^F-FET MicroPET combined with computed tomography (CT) were used to monitor tumour growth. Tumour take (TT) was considered as a FET T/B ratio above 1.2 (described in detail below). Mice with confirmed TT were subsequently divided into three groups matched according to FET T/B ratio. Treatment with B20–4.1 (B20) (5mg/kg), B20 (5mg/kg) in combination with TB403 (20 mg/kg) or 0.9% saline solution as control was administered intraperitoneally (i.p.) twice a week to the three groups, respectively.

B20 is like Bevacizumab an antibody against VEGF-A, which unlike Bevacizumab has affinity for both the human and the murine VEGF-A [21, 22]. TB403 (RO5323441) is a humanized monoclonal antibody that binds to both PlGF-1 and PlGF-2 and has affinity for both the murine and the human PlGF-2 [23]. B20 and TB403 were kindly provided by Roche (pRED oncology). Treatment was initiated the day after the ^18^F-FET PET confirming TT (the baseline scan), and the treatment response was monitored after one and two weeks. Survival was the primary end-point, and the survival time was the number of days from confirmed TT until xenografts were sacrificed according to a predefined assessment score (S1 Table). Subsequently, the brains were removed from the cranial cavity and used for immunohistochemistry (IHC). Additionally, half of the xenograft tumour was isolated from four mice from each treatment group and used for quantitative real-time polymerase chain reaction (qPCR).

### MicroPET/CT imaging

^18^F-FET was acquired from routine weekly production for clinical use (Rigshospitalet, Copenhagen, Denmark) as previously described [15]. Mice were anaesthetized with Hypnorm/Midazolam (1ml/100g bodyweight) and injected with 7.7±0.2 MBq ^18^F-FET intravenously (i.v) in the tail vein. In order to prevent hypothermia, mice were placed on an electrical heating-pad during a 10 min PET acquisition, 20–30 minutes post injection of ^18^F-FET. MicroPET Focus 120 (Siemens Medical Solutions, Malvern, USA) was used for the acquisition of the emission data (see S1 File for details). The Inveon Research Workplace (IRW) software (Siemens Medical Solutions) was used for co-registration of MicroPET and MicroCT images. At the location of maximum tracer uptake in the tumour a 3D spherical region of interest (ROI) was placed (ROI_T_). In the contralateral normal hemisphere a 4 mm^3^ spherical ROI was drawn (ROI_B_). ^18^F-FET uptake was expressed as maximum standardized uptake values (SUV_max_) in ROI_T,_ as a SUV_max_ T/B ratio ((SUV_max_ in ROI_T_)/(SUV_max_ in ROI_B_)) and as a SUV_mean_ T/B ratio ((SUV_max_ in ROI_T_)/(SUV_mean_ in ROI_B_)).

### Bioluminescence imaging

Groups of two to three mice were injected i.p. with 150 mg/kg D-luciferin in phosphate-buffered saline (Perkin Elmer, USA). Subsequently, mice were anesthetized using 2% isoflurane and placed in the IVIS Lumina XR optical imaging system (Caliper Life Sciences, Perkin Elmer, USA), at approximately 5 minutes after D-luciferin injection. Acquisition time was adjusted to optimize the signal without saturating the image while field of view and F-stop were kept constant. Scanning was continued until the peak signal was captured for each mouse. The *Living Image 4.3.2* software on the IVIS system was used for image analysis. A two-dimensional ROI at a fixed size was manually drawn covering the entire skull of the mouse, and total photon flux (photons/sec) in the ROI was measured.

### Magnetic resonance imaging (MRI)

MRI experiments were performed on a Bruker Biospec 7.0 (Bruker Biospin, Ettlingen, Germany). Mice were anaesthetized using 2% isoflurane and a water heating system, combined with a rectal thermometer, was used in order to maintain body temperature at 36.5–37.5°C. A 30-mm surface coil was fixed covering the skull of the mouse. A TurboRare T2w protocol was used for generation of transverse and coronal images. 8 transverse slices and 12 coronal slices with a thickness of 0.5 mm were acquired using a repetition time (TR) of 2500 ms and an echo time (TE) of 33 ms. The total scan time was 5 minutes and 20 seconds for each orientation. A field of view of 20×20 mm was chosen and sampled into a matrix size of 256×256 mm resulting in a spatial resolution of 0.078. The MRI images were then transferred in DICOM format into the Inveon software (Siemens Medical Solutions) for image analysis. ROIs covering the total tumour area were manually drawn on each slice and a tumour volume was obtained by interpolating the ROIs from all transverse images and the coronal images, respectively. The total tumour volume was calculated as the mean of the tumour volume in the transverse and coronal images.

### Immunohistochemistry

Intact brains were fixed in 4% paraformaldehyde for 24–48 hours at 4°C followed by incubation in 70% ethanol. Subsequently, brains were divided in two by coronal cutting in the incision site, although in the 12 mice which were used for qPCR, the brains were divided before fixation. IHC was performed on formalin-fixed paraffin-embedded tissue, and histological sections (4µM) were stained with hematoxylin and eosin (HE) for normal histological evaluation and with antibodies detecting Ki67 and CD31. All IHC stainings were performed manually and according to the manufacturer’s instruction. Primary antibodies used: CD31 (detecting both human and murine CD31, diluted 1:50, Abcam, Cambridge, UK) and Ki67 (detecting human Ki67, diluted 1:100, Abcam, Cambridge, UK).

The AxioScan Z1 slide scanner and the software ZEN 2012 (Carl Zeiss Microscopy, Germany) were used for IHC analysis. MVD was analysed treatment-blinded by capturing 4 images at a magnification of ×20 (pixel size: 0.44×0.44 µm, image size: 610×400 µm) covering the regions where the MVD was highest. Subsequently, the micro-vessels were counted manually on the computer screen using Image J 1.47 software. MVD (micro-vessels/mm^2^) of the specimens was estimated as a mean of the MVD in the four analysed regions. The online available image analysis software ImmunoRatio [24] was used for quantification of the Ki67 proliferation index (percent of DAB-staining area out of the total area). Treatment-blinded and depending on the tumour size, seven to ten images were captured at the magnification of ×40 (pixel size: 0.22×0.22 µm, image size: 310×200 µm) covering the regions in which Ki67 staining was particularly prevalent in order to avoid necrotic areas. According to the web-application, camera settings and staining intensity was evaluated and found to be acceptable. The hematoxylin and DAB thresholds were manually adjusted, and results were interpreted with the pseudo-coloured images and the original image. The Ki67 proliferation index was estimated as a mean of the Ki67 proliferation index in the 3 regions with the highest Ki67 proliferative index on the assumption that it best represents the proliferation potential of the tumour.

### Quantitative Real–time PCR

After resection, tumour specimens from xenografts and patients were snap-frozen and stored in liquid nitrogen. Total RNA from patient specimens was isolated using Trizol reagent (Gibco BRL 15596–018) and Qiagen TissueLyser before RNA purification with the RNeasy Mini kit (Qiagen, Denmark). Total RNA from xenograft tumours was isolated using RNAlater- ICE (ambion), RNAzolRT (Molecular Research Center Inc., USA) and PrecellysR-24 (Bertin Techmologies, France) for tissue homogenization. RNA measurements, reverse transcription (RT), primer design and pPCR were performed as previously described [15]. In short, the VEGFR-1 primers were purchased from DNA technology A/S (DNA technology, Denmark), and all other primers were purchased from Sigma-Aldrich (Sigma-Aldrich, USA) and designed to be human-specific. The Brilliant SYBRGreen QPCR Master Mix (Stratagene) was used and gene expression was quantified on the Mx300P real-time PCR system (Stratagene). The following thermal profile was used: denaturation for 10 minutes at 95°C followed by 45 cycles of 30 seconds denaturation at 95°C, primer annealing for 1 minute at 60°C and 1 minute extension at 72°C. Subsequently, the PCR product was denatured for 1 minute at 95°C followed by a ramp down to 55°C and a dissociation curve was acquired by a stepwise increase in temperature from 55°C to 95°C with steps of 0.5°C/cycle.

All samples were run in duplicates and included on the same plate using 1 μl of cDNA. To each plate a no-template control (NTC) was included. No reverse transcription control (NoRT) for all samples was tested using the reference genes and PlGF. Quantification of results was based on the computation of target quantification cycle (Cq) values and reference gene Cq values in the qbasePLUS 2.6.1 software (Biogazelle NV, Belgium) [25]. Genes of interest (GOI) were normalized to the arithmetic mean of the expression of the two reference genes peptidylprolyl isomerase A (PPIA) and topoisomerase 1 (TOP1) with a reference target stability of 0.82 (M-value) and 0.28 (CV-value). A default amplification efficacy of 100% was used as all assays were optimized to have efficiencies between 90% and 110%. Results were reported as normalized relative quantities (NRQs).

Primer sequences were PPIA-FP: 5’-cggatttgatcatttggtg-3’, PPIA-RP: 5’ccagacaacacacaagac-3’, TOP1-FP: 5’-agaggcattgttagtttagtg-3’, TOP1-RP: 5’-cctacagttgattaaaagggaa-3’, PlGF-FP: 5’-ctcacactttgccatttg-3’, PlGF-RP: 5’-actctgtatgtgtctcttag-3’, VEGFR1-FP: 5’-ggctctgtggaaattcagc-3’, VEGFR1-RP: 5’-gctcacactgctcatccaaa-3’. Isolation of RNA, primer design and quantitative Real-time PCR detecting PlGF and VEGFR-1 expression was performed (described in S5).

### Statistical analysis

All statistical analyses were performed using GraphPad Prism version 6.0 for MAC OS X (GraphPad Software Inc., USA). All comparisons between the treatment groups were performed using one-way ANOVA assuming Gaussian distribution. P values were adjusted by Sidak’s multiple comparisons test. All data are presented as mean ± SEM (standard error of mean) if not stated otherwise. P < 0.05 was considered statistically significant. Survival analysis was performed using the Kaplan-Meier method and the log-rank test.

## Results

### Tumour model characteristics

A total of 35 mice were injected orthotopically with GBM017_LUC cells. Three weeks after tumour cell injection, 32 mice had confirmed TT with a FET SUV_max_ T/B ratio above 1.2. One mouse died from anaesthesia before the first evaluation scan was performed, and therefore, TT status was unknown. The remaining 2 mice had confirmed TT 4 weeks after orthotopic injection. A total of 34 mice were included in the treatment study of which 13 mice were included in the control group, 11 mice in the B20 group and 10 mice in the B20+TB403 group. One mouse from the B20+TB403 group was excluded from the final image analysis as tumour symptoms hindered evaluation after one week of treatment.

### ^18^F-FET PET imaging of orthotopic GBM xenografts

**Figure 1:**
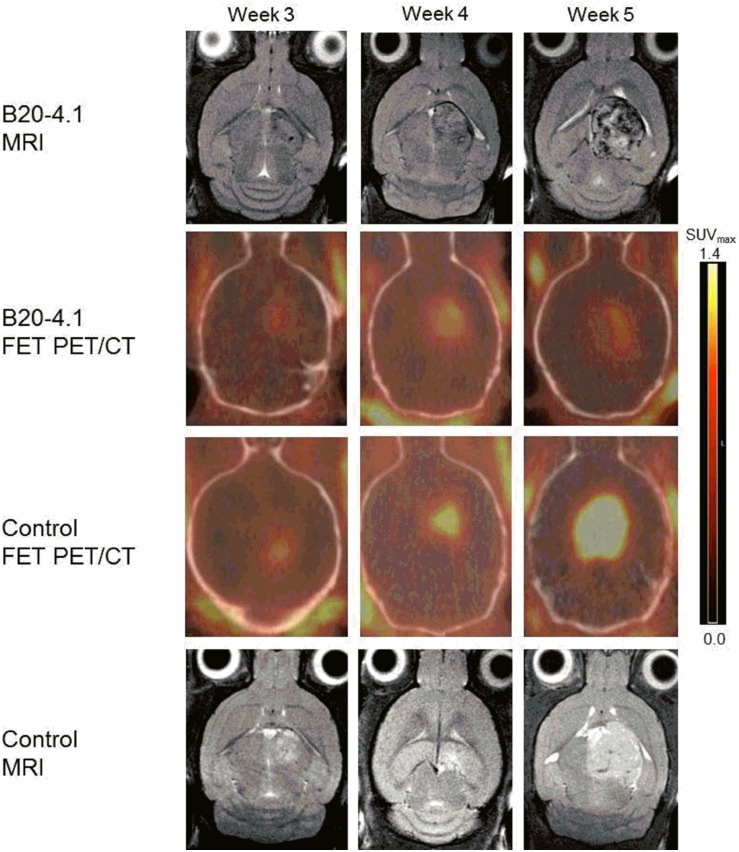
Illustration of tumour progression and treatment response. T2w-MRI and fused ^18^F-FET MicroPET/CT images showing tumour progression 3–5 weeks after tumour cell injection. Transverse views through the brain of a mouse from the B20–4.1 group and a mouse from the control group. Scale bar: 0–1.4 SUV_max_

**Figure 2:**
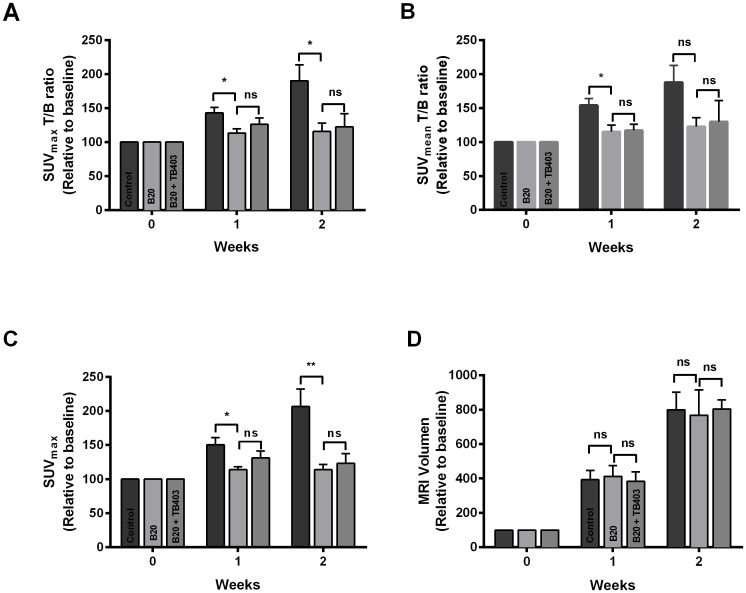
^18^F-FET MicroPET/CT to monitor treatment response. ^18^F-FET uptake in the treatment groups expressed as A) SUV_max_ T/B ratio, B) SUV_mean_ T/B ratio and C) SUV_max_. D) MRI volume in the treatment groups. All values are expressed as mean ± SEM relative to baseline (week 0) after 1 week of treatment in the control (n = 13), the B20 group (n = 11) and in the B20+TB403 (n = 9); and after 2 weeks of treatment in the control (n = 5), the B20 group (n = 8) and in the B20+TB403 group (n = 6). *p<0.05, **p<0.01

Fig. 1 shows representative ^18^F-FET MicroPET/CT and T2w-MRI of an orthotopic GBM017_LUC tumour from the control and the B20 group. Here, a difference in the tumour uptake of ^18^F-FET between the B20 and the control mouse is clear, as a higher signal is observed for the control group. In contrast, T2w-MRI is more difficult to assess for possible differences in tumour size. In order to investigate whether the ^18^F-FET MicroPET/CT could be used quantitatively to detect a response to treatment, in this case B20 and B20+TB403, the ^18^F-FET uptake in the three groups (relative to baseline) was plotted versus time following TT (Fig. 2). The relative SUV_max_ T/B ratio was significantly higher after one week of treatment (143±8, n = 13 vs. 114±6, n = 11; p = 0.019) and after two weeks of treatment (190±24, n = 5 vs. 116±12, n = 8; p = 0.018) in the control group as compared to the B20 group (Fig. 2A). In contrast, there was no significant difference in the SUV_max_ T/B ratio between the B20 and the B20+TB403 group neither after one week of treatment (114±6, n = 11 vs. 126±9, n = 9; p = 0.49) nor after two weeks of treatment (116±12, n = 8 vs. 123±19, n = 6; p = 0.95) (Fig. 2A). When we used the SUV_mean_ T/B ratio to evaluate the treatment response, we found a significant difference between the control group and the B20 group after one week of treatment (154±10, n = 13 vs. 115±10, n = 11; p = 0.013), but after two weeks of treatment the difference was not significant (188±25, n = 5 vs. 123±13, n = 8; p = 0.11) (Fig. 2B). In line with the results using the SUV_max_ T/B ratio to quantify FET uptake, we did not find a significant difference in the SUV_mean_ T/B ratio between the B20 and the B20-TB403 group neither after one week of treatment (115±10, n = 11 vs. 117±9, n = 9; p = 0.99) nor after two weeks of treatment (123±13, n = 8 vs. 130±31, n = 6; p = 0.97) (Fig. 2B).

We then evaluated whether we also were able to detect a treatment response by evaluating the ^18^F-FET uptake expressed as SUV_max_. In Fig. 2C, the mean SUV_max_ of ^18^F-FET was plotted versus time after TT in the treatment groups. In the B20 group the mean SUV_max_ was significantly lower after one week of treatment (114±4, n = 11 vs. 150±11, n = 13; p = 0.012) and after two weeks of treatment (114±8, n = 8 vs. 207±26, n = 5; p = 0.001) as compared to the control group. Again, when comparing the B20 and the B20+TB403 groups, the results from the evaluation of the T/B ratio were confirmed; as we did not find any significant difference in mean SUV_max_ neither after one week of treatment (114±4, n = 11 vs. 131±10, n = 9; p = 0.38) nor after two weeks of treatment (114±8, n = 8 vs. 123±14, n = 6; p = 0.88).

### MRI of orthotopic GBM xenografts

As we were able to detect a treatment response towards B20 monotherapy using ^18^F-FET MicroPET, we wanted to evaluate if this treatment response was reflected in a difference in the anatomical tumour volume as measured by T2w-MRI. However, we found no difference in total tumour volume in the B20 group as compared to the control group neither after one week of treatment (412±63, n = 11 vs. 394±54, n = 12, p = 0.97) nor after two weeks of treatment (768±147, n = 8 vs. 800±103, n = 5, p = 0.98), (Fig. 2D). Furthermore, we found no difference between the B20 and the B20+TB403 groups neither after one week (412±63, n = 11 vs. 383±54, n = 9, p = 0.93) nor after two weeks of treatment (768±147, n = 8 vs. 804±52, n = 6, p = 0.97), (Fig. 2D).

### Bioluminescence imaging of orthotopic GBM xenografts

**Figure 3:**
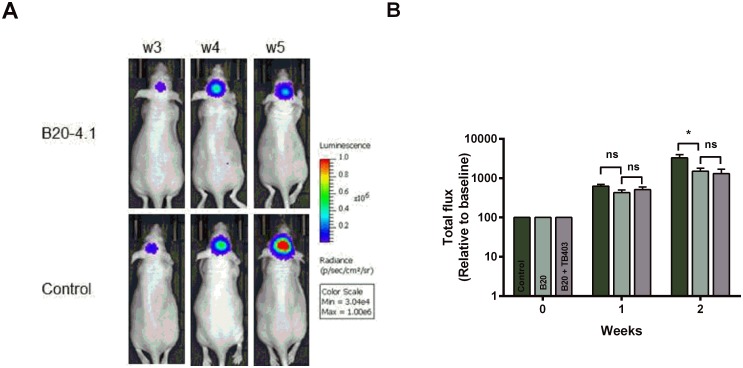
Bioluminescence imaging to monitor treatment response. A) Representative images of bioluminescence 3–5 weeks after tumour cell injection showing tumour progression in a B20–4.1 mouse and a control mouse. B) Quantification of total flux relative to baseline (week 0). Values are expressed as mean ± SEM after one week of treatment in the control (n = 13), the B20 group (n = 11) and the B20+TB403 group (n = 8); and after two weeks of treatment in the control (n = 9), the B20 (n = 9) and the B20+TB403 group (n = 6). *p<0.05

As results from the FET MicroPET and T2w-MRI were conflicting, we evaluated results from quantification of the BLI images, where total flux is a measure of viable tumour cells. Fig. 3A shows BLI images of a representative xenograft from the B20 and from the control group for visual comparison, while Fig. 3B shows the mean total flux between the three treatment groups. In line with results from the ^18^F-FET MicroPET, we found a significantly lower mean total flux in the B20 group after two weeks of treatment as compared to the control group (1507±296, n = 9 vs. 3296±685, n = 9, p = 0.03). Contrary, after one week of treatment, there was only a trend towards a significant difference between the B20 and the control group (431±70, n = 11 vs. 629±65, n = 13, p = 0.098). When comparing the B20 group with the B20+TB403, no significant difference was detected neither after one week of treatment (431±70, n = 11 vs. 512±88, n = 8, p = 0.71) nor after two weeks of treatment (1507±296, n = 9 vs. 1305±390, n = 6, p = 0.96), and the results thereby confirmed the ^18^F-FET MicroPET data.

### Treatment effect on survival

**Figure 4:**
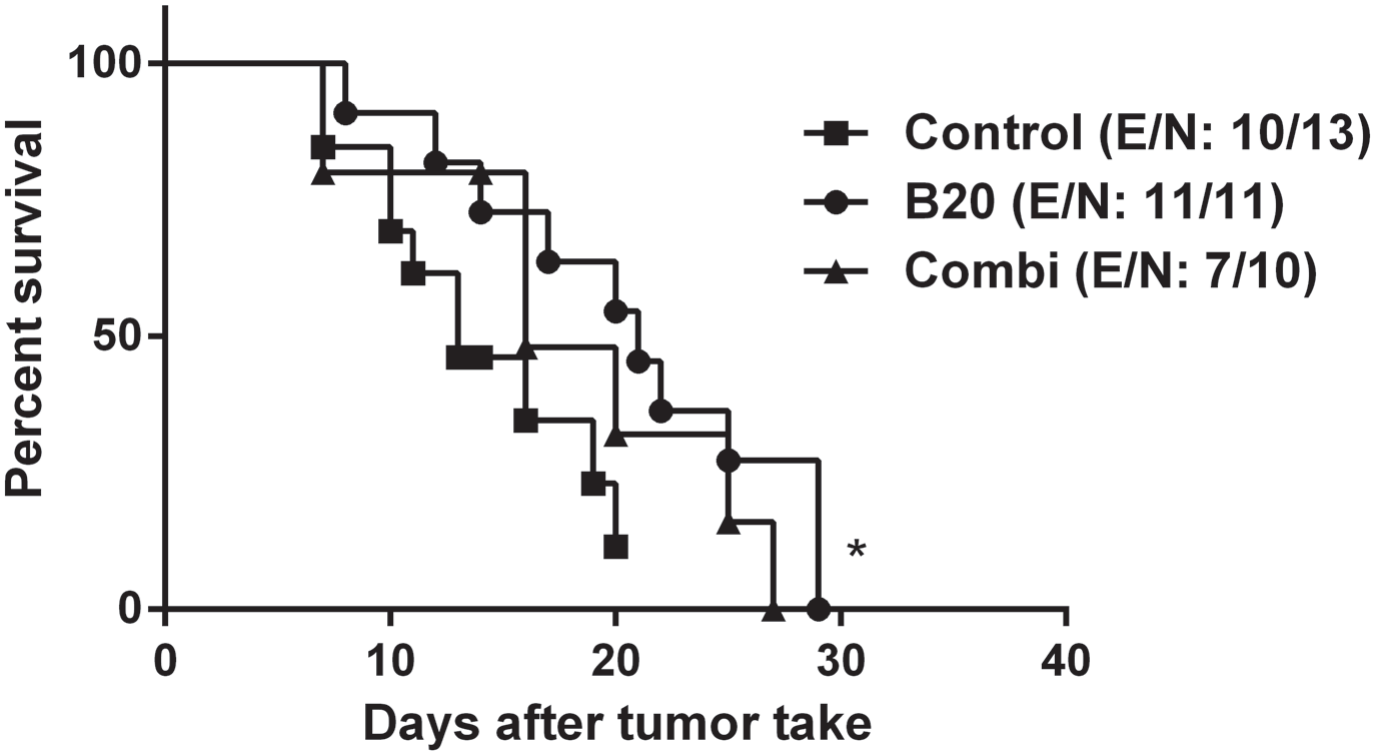
Survival analysis. Kaplan Meier survival curves from tumour take. Control group vs. B20 group, 13 vs. 21 days; p = 0.04; HR = 0.5; 95% CI: 0.13–0.82 (determined by log-rank test). E: events, N: number of animals

In Fig. 4, Kaplan-Meier survival curves are shown. Comparison of the B20 group with the control group using Log-rank test, showed a significant increase in mean survival time (13 vs. 21 days; p = 0.04; HR = 0.5; 95% CI: 0.13–0.82), while no significant difference in survival was found between the B20 and the B20+TB403 group (21 vs. 16; p = 0.30; HR = 0.63; 95% CI: 0.2–1.6). 6 mice were censored as they died from anaesthesia before the criteria for euthanasia were fulfilled.

### Ki67 labelling index and Micro-Vessel Density (MVD)

**Figure 5:**
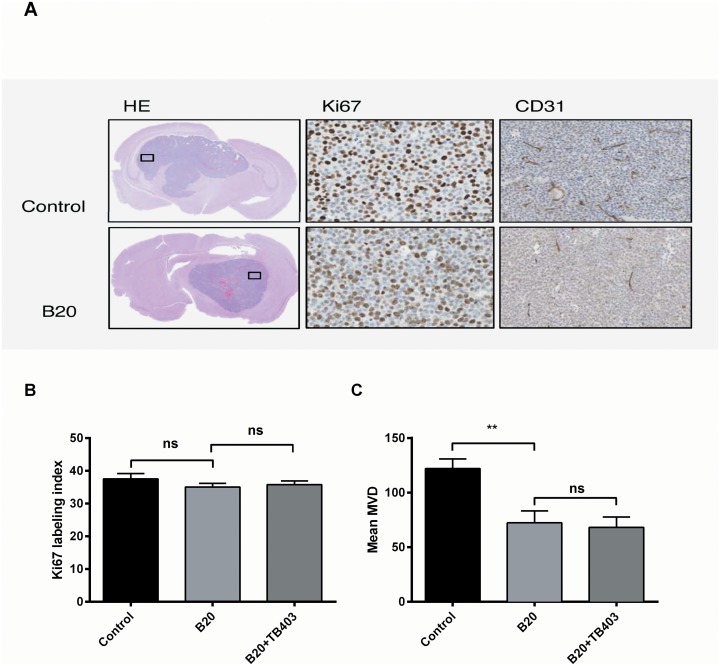
Immunohistochemistry of xenograft tumours. A) Representative IHC images of HE and high magnification of Ki67 (40x) and CD31 (20x). B) Ki67 proliferation index and C) MVD in the control group (n = 11), the B20 group (n = 10) and the B20+TB403 group (n = 7). Mean ± SEM, **p<0.01

As we wanted to investigate if the observed changes in FET uptake, BLI and survival corresponded to molecular markers of proliferation or MVD we performed IHC. Representative pictures from HE, Ki67 and CD31 stained tissue sections are shown in Fig. 5A, while Fig. 5B and 5C shows results from quantification of Ki67 and MVD. In contrast to the results from FET Micro/PET, BLI and survival analysis, but in line with the MRI findings, we found no difference in the Ki67 labelling index (estimated from the three hotspot regions) neither in the B20 group as compared to the control group (35±1, n = 9 vs. 37.5±2; n = 11; p = 0.34), (Fig. 5B) nor in the B20 group as compared to the B20+TB403 group (35±1, n = 9 vs. 36.5±1; n = 7; p = 0.93). When we compared the treatment groups, using a Ki67 labelling index estimated from 7–10 regions in each tumour, the results were similar (not shown). However, we found a significant lower MVD in the B20 group as compared to the control group (72±11, n = 10 vs. 122±9 n = 11; p = 0.002), while the MVD was comparable in the B20 and the B20+TB403 groups (72±11, n = 10 vs. 68±9 n = 7; p>0.99 (Fig. 5C).

### Quantification of PlGF and VEGFR-1 mRNA expression in xenografts and patient tumours

**Figure 6:**
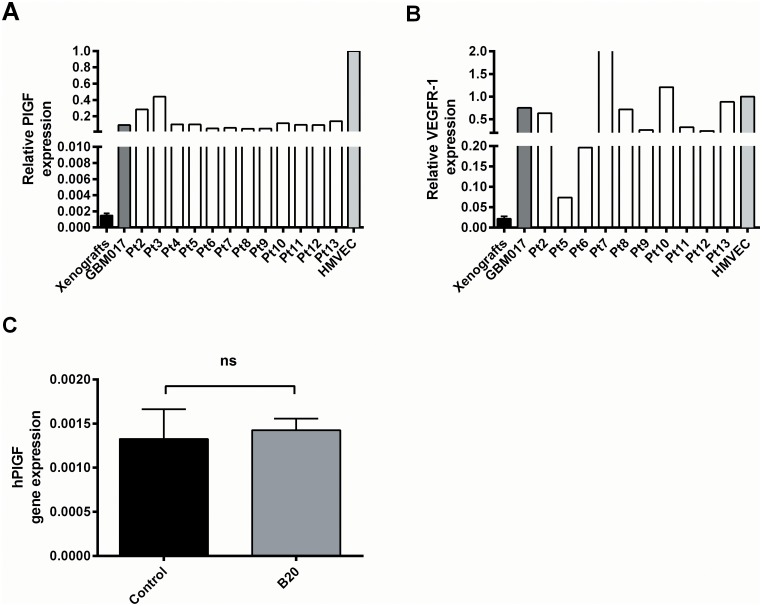
Gene expression analysis of xenograft tumours. Gene expression of A) PlGF and B) VEGFR-1 in xenografts and a panel of GBM patients including patient GBM017. Values in xenografts are expressed as mean ± SEM (n = 12). C) Xenograft gene expression of PlGF in the control group (n = 4), the B20 group (n = 4). All genes are normalized to reference genes and are relative to human HMVEC

In order to investigate if the lack of an additional anti-tumour effect of anti-PlGF could be related to the expression level of PlGF and/or VEGFR-1 in the tumour cells, we performed qPCR for PlGF and VEGFR-1 in a small subset of the xenograft tumours and compared the expression to a panel of 13 GBM patients. The gene expression of the tumour samples was normalized to reference genes and scaled to the gene expression in HMVEC, which was adjusted to 1. The relative gene expression is shown in Fig. 6A and 6B. As compared to the panel of GBM patients, including the patient tumour from which the GBM_CHP017 cells were established, we found a much lower expression of both PlGF and VEGFR-1 in the xenografts. In addition, no significant difference in the gene expression of PlGF between the B20 group and the control group xenografts was observed (Fig. 6C), showing that the gene expression of PlGF was unchanged in response to anti-VEGF (B20) treatment.

## Discussion

In the present study, we have used ^18^F-FET MicroPET in combination with MRI to evaluate a treatment response towards anti-VEGF (B20) monotherapy and towards combined treatment with anti-PlGF (TB403) and anti-VEGF (B20). In contradiction to our hypothesis, we found no significant difference in ^18^F-FET uptake, MRI, BLI, Ki67 proliferative index, MVD or survival between the B20 and the B20+TB403 groups. Thus, the combination of anti-PlGF and anti-VEGF did not have an additive effect on tumour growth in the GBM017_LUC orthotopic tumour model used in the present study. The efficacy of anti-PlGF has been related to the expression of PlGF and its receptor VEGFR-1 [26]. We found a very low expression of both PlGF and VEGFR-1 in the xenografts as compared to the patient tumours, which obviously could explain the lack of efficacy of anti-PlGF. However, it is striking that the tumour specimens from the GBM017 glioma patients had a much higher expression of PlGF and VEGFR-1 as compared to the xenografts. A possible explanation for this is that PlGF and VEGFR-1 observed in the patient tumour is derived from cancer associated stroma cells or endothelial cells and not from the cancer cells. If PlGF and VEGFR-1 primarily is derived from stromal cells in the orthotopic xenograft tumours it is of murine origin and therefore not detectable with the human specific primers. This explanation is supported by recently published data in prostate cancer where PlGF is overexpressed in fibroblasts and undetectable in the prostate cancer cells [27]. Regardless whether there was an undetected stromal contribution of PlGF and/or VEGFR-1 in the present study, we did not detect an effect of adding anti-PlGF therapy to anti-VEGF monotherapy. This is in line with a recent Phase 1–2 study of Bevacizumab and TB403 in patients with recurrent GBM. Response data, time to progression, pharmacodynamics data including MRI did not indicate any additional activity of TB403 compared to Bevacizumab monotheraphy [28]. However, a study has demonstrated especially high expression levels of PlGF in selected hypervascular gliomas [29]. Among the patient tumours examined in this study, we also found two tumours (pt. 2 and pt. 3) with a PlGF level considerable higher than the other patient samples. This supports that anti-PlGF could be effective in certain glioma patients and advocate further investigation of anti-PlGF efficacy.

Others have demonstrated an up-regulation of the gene expression of PlGF in response to anti-VEGF treatment [30–32]. In contradiction to these results, we did not find a significant difference between the B20 and the control group. However, we interpret these results with caution given the small sample size, the use of human specific primers and the very low PlGF gene expression in the xenografts.

In the present study, we additionally have investigated the feasibility of *in vivo* imaging and noninvasively response assessment using ^18^F-FET Micro/PET and MRI in orthotopic GBM xenografts. We found that it is possible to detect a tumour response towards anti-VEGF (B20) using ^18^F-FET already after one week of treatment. This is in line with our previous studies where we used ^18^F-FET MicroPET to evaluate response towards Irinotecan [15] and B20 (unpublished data) in another orthotopic GBM model. In the present study, we also used T2w-MRI and demonstrated that the observed tumour response (and increased survival) was not reflected in measurable volume changes as depicted by T2w-MRI. In clinical practice, response evaluation is performed according to the Response Assessment in Neuro-Oncology (RANO) criteria [33] and it includes contrast-enhanced T1 weighted-(T1w)-MRI in conjunction with T2w/fluid-attenuated inversion recovery (FLAIR). Gadolinium contrast was not used in the present study and it is possible that T1w-MRI using contrast would have helped to differentiate between surrounding oedema and true tumour margins and therefore would have resulted in significant changes in the tumour volume. However, a lack of correlation between tumour volume and contrast enhancement was recently demonstrated in a pre-clinical study, and it was concluded that a reduction in contrast enhancement was not an accurate reflection of tumour growth in response to anti-angingenic treatment [34], which is in contradiction to this reasoning. It is likely that small changes in viable tumour cells are difficult to visualize on anatomical MRI scans where e.g. necrosis often is a part of the detected anatomical tumour volume. As such, our results therefore indicate that ^18^F-FET PET is superior to MRI for the detection of anti-VEGF efficacy in GBM xenograft models. This is in line with several clinical studies, where there is increasing evidence for the use of ^18^F-FET PET as an addition to MRI as ^18^F-FET PET adds complementary information about tumour growth [16–19].

In addition to MicroPET and MRI, we also evaluated tumour development using BLI. It is widely accepted that the BLI has a higher sensitivity than MicroPET and MRI [35], and as such, intracranial gliomas are detectable at a smaller tumour-size using BLI as compared to FET Micro/PET. Therefore it was surprising that we only detected a significant difference in BLI after two weeks of treatment while there after one week of treatment only was a trend towards significance. Although BLI is a very sensitive and well-established method for assessment of tumour-cells [36, 37] several limitations have been addressed. Changes in the expression of luciferase, hypoxia, pH and different tumour location may compromise the reliability of BLI as a quantitative measure of viable tumour cells [36, 38]. However, at the first evaluation time (week one) it could be speculated that the difference in ^18^F-FET uptake primarily is caused by a difference in the tumour vasculature rather that a difference in the tumour mass, which would explain the unchanged BLI signal.

We evaluated the Ki67 proliferative index as a marker of proliferation and as a molecular indicator of anti-proliferative activity. In contrast to our results from the FET MicroPET, BLI and survival analysis, and therefore supportive of the MRI findings, we were unable to detect a significant difference in the Ki67 proliferative index when we performed IHC at the time of study endpoint. However, it could be speculated that we would have detected a difference in the Ki67 proliferative index if the xenograft brains were analysed at an earlier time point before the brain tumours grew too large. When analysing MVD of the xenograft tumours, we found a highly significant reduction in MVD in the B20 and B20+TB403 groups as compared to the control group. This anti-angiogenic effect is in line with previous studies were B20 and anti-VEGF treatment were evaluated [34, 39] and supportive of our results from the FET MicroPET, BLI and survival analysis. Controversy exists whether Bevacizumab has direct anti-cancer activity or if the clinical benefit observed in glioma patients exclusively is caused by effects on the tumour vessels [39]. The latter could be an explanation for the decreased MVD and unchanged Ki67 proliferative index observed in the present study.

Taken together, results from the FET MicroPET, BLI and MVD analysis were all in agreement with the increased survival observed in the B20 treated groups. Thus, as observed in patients, this study shows that ^18^F-MicroFET PET compared to T2w-MicroMRI adds valuable complementary information about tumour growth in orthotopic glioma xenografts. Hence, ^18^F-FET MicroPET could be a valuable imaging modality for preclinical evaluation of new therapies in orthotopic GBM xenograft models. However, the technique should be combined with GBM models that reflect the diversity of GBM tumours in patients, in order to obtain preclinical results that are transferrable to the clinic.

## Conclusion

In orthotopic GBM xenografts anti-VEGF monotherapy increased survival and reduced ^18^F-FET uptake after one and two weeks of treatment without significant changes in anatomical T2w-MRI volume. This study demonstrates that anti-angiogenic response assessment using ^18^F-FET MicroPET is feasible and superior to T2w-MRI. Furthermore, the combination of anti-VEGF and anti-PlGF did not result in an additive effect on tumour growth or survival. As such, this study supports the additional use of ^18^F-FET PET in the evaluation of patients with GBM and in preclinical studies as ^18^F-FET PET might be an early and non-invasive biomarker for detection of anti-angiogenic treatment response or failure.

## Supporting Information

S1 Table Monitoring and euthanasia of glioblastoma xenografts. (DOC)

S1 File MicroPET/CT acquisition details. (DOCX)
