# Supplementary material for: 18F-FET MicroPET and MicroMRI for Anti-VEGF and Anti-PlGF Response Assessment in an Orthotopic Murine Model of Human Glioblastoma
Source: PLoS One. 2015 Feb 13;10(2):e0115315. doi: 10.1371/journal.pone.0115315 (PMC4332497; doi:10.1371/journal.pone.0115315)
Supplement: S1 Table — (DOC) [file pone.0115315.s001.doc]

**Table S1. Monitoring and euthanasia of glioblastoma xenografts**

| Variable | Score |
| --- | --- |
| **Body Weight Changes** |  |
| <20% | 0 |
| >20% | 3 |
| **Physical Appearance** |  |
| Normal | 0 |
| Lack of grooming | 1 |
| Small bites or scratches. Nasal/ocular discharge | 2 |
| Serious bites or scratches. Abnormal posture, limb, tremor etc. | 3 |
| **Unprovoked Behavior** |  |
| Normal | 0 |
| Minor changes | 1 |
| Abnormal, reduced mobility, decreased alertness, inactive | 2 |
| Unsolicited vocalizations, self-mutilation, either very restless or immobile | 3 |
| **Behavioral Responses to External Stimuli** |  |
| Normal | 0 |
| Minor depression/exaggeration of response | 1 |
| Moderately abnormal responses | 2 |
| Violent reactions or comatose | 3 |
| **Occipital Tumor** |  |
| None | 0 |
| Palpable | 1 |
|  |  |
|  |  |
|  |  |
| **TOTAL SCORE** |  |

Euthanasia of the mouse at

- Total score > 5, or
- A score of 3 in any one variable, regardless of the total score.
