# Supplementary material for: 18F-FET MicroPET and MicroMRI for Anti-VEGF and Anti-PlGF Response Assessment in an Orthotopic Murine Model of Human Glioblastoma
Source: PLoS One. 2015 Feb 13;10(2):e0115315. doi: 10.1371/journal.pone.0115315 (PMC4332497; doi:10.1371/journal.pone.0115315)
Supplement: S1 File — (DOCX) [file pone.0115315.s002.docx]

**File S1. MicroPET/CT acquisition details**

An energy window of 350-650 keV and a time resolution of 6 ns were set up for the acquisition. The acquired datasets were stored in Listmode before arranging into 2D sinograms. The sinograms were then reconstructed into 256×256×95 image matrices with a voxel size of 0.3×0.3×0.8 mm3 using the maximum a posteriori (MAP) reconstruction algorithm. The intrinsic PET resolution was 1.2 mm full-width at half-maximum. Scatter and attenuation correction were not applied. The quantification unit was provided in Bq/ml. Subsequently, a four minutes CT scan was acquired in order to get anatomical information using a small animal CT scanner (MicroCAT II system, Siemens Medical Solutions). To quantify the ^18^F-FET uptake, the standardized uptake values (SUVs) were calculated from the equation: SUV= CT / (Dinj x W). CT is the radioactivity in tissue with the unit Bq/ml, Dinj is the injected dose and W is the weight of the mouse. SUV_max_ was calculated from the voxel with the highest tracer concentration in the ROI. SUV_mean_ was calculated as the mean radioactivity in the ROI.
